# Aseptic bone-flap resorption after cranioplasty - incidence and risk factors

**DOI:** 10.1371/journal.pone.0228009

**Published:** 2020-01-30

**Authors:** Ali Rashidi, I. Erol Sandalcioglu, Michael Luchtmann

**Affiliations:** Department of Neurosurgery, Medical Faculty, Otto-von-Guericke University Magdeburg, Magdeburg, Germany; Ohio State University, UNITED STATES

## Abstract

**Objective:**

One of the common complications occurring after cranioplasty (CP) is aseptic bone-flap resorption (ABFR). Reoperation necessary because of the development of ABFR can lead to unfavorable complications during subsequent surgery using a synthetic skull implant, and also necessarily leads to higher costs. The aim of this study is to identify prognostic factors that may help to predict the development of ABFR.

**Methods:**

In this study, 303 CP surgeries performed between 2002 and 2017 were examined retrospectively to identify factors predicting the occurrence of ABFR. A number of these factors (e.g., time lapse between decompressive craniectomy (DC) and CP, bone-flap size, specific laboratory signs, and the reason for the original DC) were analyzed as possibly influencing the risk of developing ABFR.

**Results:**

ABFR of an autologous bone flap that subsequently required a CP with synthetic skull implants occurred in 10 of 303 patients (3.0%). CP timing and patients' Karnofsky Performance Scores (KPS) (p = 0.008; p = 0.012) were identified as significant factors with an impact on the development of ABRF. Age did not reveal a significant value, but statistical analysis shows a clear trend. The younger the age, the more likely it was that an ABFR would develop.

**Conclusion:**

The risk of ABFR lessens the longer the period of time elapsed between DC and CP. Age does not reveal a significant value, but statistical analysis shows that there is a clear trend.

## Introduction

Decompressive craniectomy (DC) is performed to reduce refractory increased intracranial pressure e.g., pressure due to intracerebral hemorrhage, traumatic brain injury, or ischemic stroke[1–3]. After the resolution of a brain edema and/or reduction of intracranial pressure, following cranioplasty (CP) may lead to improved cerebral blood circulation and a better neurosurgical outcome,[1, 3–8] not merely in terms of cosmetic appearance but, more important, the overall protection of the brain. Additionally, positive effects on the hydrodynamics and metabolism of cerebrospinal fluid have also been reported [8–10].

Autologous bone flap harvested at the time of a DC, as well as synthetic materials including porous polyethylene, methyl methacrylate, titanium, hydroxyapatite, ceramics, and osteoconductive bioresorbable materials, are frequently used to perform CP [11–15]. Autologous bone material has several advantages over synthetic materials, including its perfect shape, lower rejection response rate, higher patient acceptance rate, and low cost, making it an ideal material for the reconstruction of cranial defects [13, 15–19]. However, despite these clear advantages, reimplantation of the autologous bone flap involves some risks. Aseptic bone-flap resorption (ABFR) is one of the most common postoperative complications to occur after a CP [13]. A subsequent ABFR may require secondary surgery with the associated risks of neurological deterioration and prolonged hospital stay, and consequently an increased economic burden. The purpose of this study was to identify risk factors associated with the occurrence of ABFR. We focused our research on cases of ABFR marked by severe resorption, where the bone flap lost its supporting function and thus required reconstruction of the skull using synthetic implants.

## Materials and methods

In this study, we retrospectively examined CPs performed at our neurosurgical department, between 2002 and 2017. All data were collected from the hospital information system and fully anonymized prior to statistical analysis. The presented study was approved by the Local Ethics Committee of the University of Magdeburg in compliance with national legislation and the Code of Ethical Principles for Medical Research Involving Human Subjects of the World Medical Association (Declaration of Helsinki). Due to the retrospective character of the study the requirement for informed consent was waived.

A total of 329 patients (195 men and 134 women) underwent a CP during that time period. In 303 of 329 patients, the autologous bone flap was reimplanted. In the remaining 26 cases, a synthetic skull implant was inserted. The main aim of this study was to investigate risk factors affecting the incidence of ABFR after CP. These risk factors were derived from the recent literature (see Table 4). Additionally, the analysis was extended to include factors which have not yet been taken into account. The following parameters were evaluated:

- CP timing [months]
- Age of patient [years]
- Size of bone flap [cm^2^]
- Number of bone-flap parts [N]
- Karnofsky performance score (KPS)
- Cause of DC
  - Trauma
  - Stroke
  - Intracerebral hemorrhage
  - Subarachnoid hemorrhage
  - Tumor
  - Infection
  - Other
- Laboratory signs of infection
  - C-reactive protein [> 5 mg/l]
  - White blood cells [> 10.4 Gpt/l]
  - Platelet count [> 400 Gpt/l]
  - Previous and subsequent radio- and/or chemotherapy
- Diabetes mellitus
- Ventriculoperitoneal shunting (VPS) [before, simultaneous to, or after CP]
- Fixation technique (titanium clamps, miniplates, a combination of both)

In our clinical routine, Decompressive Craniectomy was performed and bone flaps were freed from adherent soft tissue residuals, they were separately packed in sterile bags and kept at -80°C under aseptic conditions. During the Cranioplasty (CP) bone flaps were removed from the freezer and thawed at room temperature. During CP, dural tack-up sutures were placed through the bone flap. Subsequently, skull implants were fixed to the skull using miniplates and/or titanium clamps. Finally, a subgaleal Jackson-Pratt drain was put in place.

The first postoperatively computed tomography (CT) was performed during the postoperative hospital stay to evaluate the correct position of the reinserted bone flap and to serve as a reference for follow-up examinations, which were usually scheduled for three months after the CP and annually thereafter. Palpable instability and changes in appearance were detected by clinical examination. The mean follow-up time was 13.2 ± 25.0 months. Aseptic bone-flap resorption was primally diagnosed by clinical examination and confirmed by radiological imaging (cranial CT).

We focused our efforts on severe cases of ABFR only, which were defined as having arrived at a stage of resorption where the bone flap had lost its supporting function and required reconstruction with synthetic skull implants. These cases correspond to type II (Aseptic bone resorption with circumscribed or complete lysis of tabula interna and externa.) of ABFR in the contemporary classification systems of Dunisch et al. [4]. The patient's condition was assessed using KPS.

### Statistical analysis

Statistical analysis was performed using the software SAS 9.4 (SAS Institute, Inc., Cary, NY, USA). Because each analysis was performed in an explorative sense, each was deliberately reviewed to the full level of significance. Every p-value ≤ 0.05 thus represents a statistically significant result. For unadjusted analyses, chi-square tests were used for categorical variables and the robust t-test (Satterthwaite) for continuous variables. To assess influence factors in multivariable analyses, the binary logistic regression model was used. This model included CP timing, specific laboratory signs of infection, and bone-flap size, all of which had been assumed to have a possible influence on the rate of infection. Estimates for pairwise odds ratios (ORs) and the corresponding 95% confidence interval based on the Wald test were given.

## Results

The CP was performed from 18–2,199 days (mean 182.3 ± 194.1 days) after the initial DC. As shown in Table 1, patients' ages were between 2 and 91 years (with a mean of 51.2 ± 17.0 years). The most common causes for the performance of a DC were traumatic brain injury (36.3%) and ischemic stroke (31.0%).

**Table 1:** Gender and cause of decompressive craniectomy.

		N	%
**Sex**	Male	179	59,1
	Female	124	40,9
**Cause of decompressive craniectomy**	Trauma	110	36,3
	Stroke	94	31,0
	Intracerebral hemorrhage	41	13,5
	Subarachnoid hemorrhage	38	12,5
	Tumor	11	3,6
	Infection	3	1,0
	Others	6	2,1

**Fig 1:**
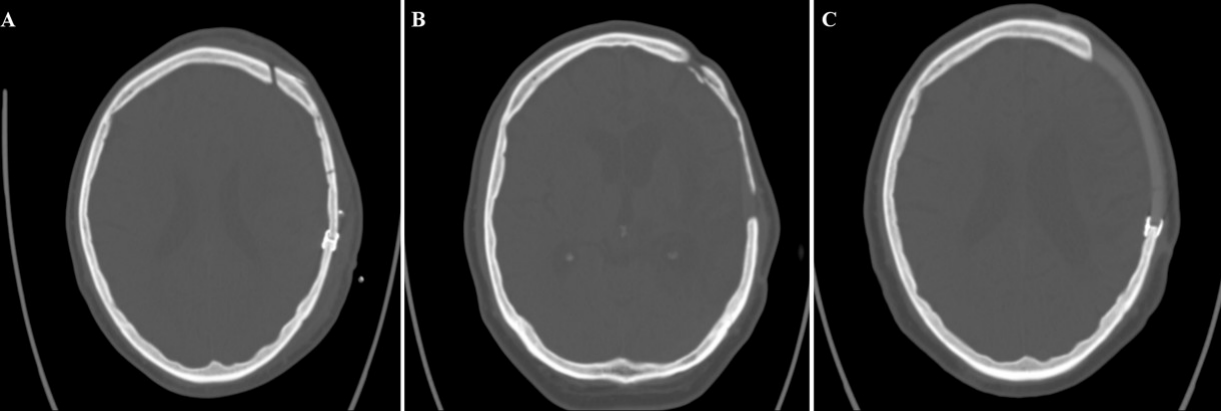
(A) Cranial computer tomography demonstrating the refixated bone flap directly after CP. (B) Same bone flap developing severe ABFR. (C) CT showing the subsequently replaced synthetic skull implant after reoperation.

ABFR of an autologous bone flap that subsequently required a CP with synthetic skull implants occurred in 10 of the 303 patients (3.0%). On average, ABFR was diagnosed after 28.0 ± 24.3 months. Fig 1 shows the typical progression of bone-flap resorption and subsequent cranioplasty using a synthetic skull implant.

Tables 2 and 3 document the results of our statistical analyses of the parameters that we originally expected to play a role in the development of ABFR and thus investigated for a possible influence. Gender did not play any role in the development of ABFR, but the KPS seems to have been a significant predictor, indicating that a higher KPS is associated with an increased risk of ABFR (p = 0.012). Our study found that CP timing had a significant influence on the development of ABFR as well. The risk of ABFR was lower the more delayed the CP was (ABFR group: 3.8±1.4 months vs. non-ABFR group: 5.3±4.1; p = 0.008). The age of the patients at the time of implantation seems to have had an influence on the risk of developing ABFR, but the statistical test fell just short of statistical significance (p = 0.056). Thirty-two patients developed hydrocephalus that required placement of ventriculoperitoneal shunting. Shunt therapy did not correlate with an increased rate of ABFR, nor did the original reason for the DC nor the fixation technique used in that surgery.

**Table 2:** Descriptive statistics and results of unadjusted tests for ABFR for categorical variables.

		Aseptic bone-flap resorption					
		Yes		No		∑	
		N	%	N	%		p-value
**Trauma**	Yes	6	60	104	35,5	110	0.113
	No	4	40	189	64,5	193	
**Laboratory signs of infection**	Yes	7	70	238	81,2	245	0.375
	No	3	30	55	18,8	58	
**Radio- and/or chemotherapy**	Yes	0	0	9	3,1	9	0.574
	No	10	100	284	96,9	294	
**Decompressive craniectomy side**	Left	5	50	146	49,8	151	0.992
	Right	5	50	147	50,2	152	
**Diabetes**	Yes	1	10	36	12,3	37	0.828
	No	9	90	257	87,7	266	
**Ventriculoperitoneal shunting**	No	10	100	261	89,1	271	0.748
	After the cranioplasty	0	0	7	2,4	7	
	Simultaneously performed	0	0	21	7,2	21	
	Before the cranioplasty	0	0	4	1,4	4	
** **	Titanium clamps	6	60	195	66,6	201	
**Fixation technique**	Miniplates	3	30	72	24,6	75	0.909
** **	Combination	1	10	26	8,8	27	

**Table 3:** Descriptive statistics and results of unadjusted tests for ABFR for continuous variables.

		Aseptic bone-flap resorption		
		Yes	No	p-value
Age [years]	N / Mean ± STD	10 / 41.0 ± 15.7	293 / 52.0 ± 16.7	0.056
Cranioplasty timing [months]	N / Mean ± STD	10 / 3.8 ± 1.4	293 / 5.3 ± 4.1	**0.008**
Size of bone flap [cm^2^]	N / Mean ± STD	10 / 81.8 ± 27.4	293 / 82.6 ± 22.7	0.932
Number of bone-flap parts	N / Mean ± STD	10 / 1.4 ± 1.0	293 / 1.2 ± 0.5	0.442
Karnofsky performance score	N / Mean ± STD	10 / 81.0 ± 21.8	293 / 59.4 ± 20.4	**0.012**

The multivariable model for analysis of the ABFR failed to show significant influence of the observed parameters (CP timing: OR = 0.525 [0.107; 2.576], p = 0.427; specific laboratory signs of infection: OR = 0.803 [0.173; 3.728], p = 0.779; and bone-flap size: OR = 0.995 [0.970; 1.021], p = 0.709. Thus, there was no evidence of the remaining variables having significantly influenced the occurrence of ABFR.

## Discussion

ABFR is one of the major complications that occurs frequently after the reimplantation of autologous bone flaps [15, 20, 21]. In the present study, the rate of ABFR was close to 3%. This incidence rate is in line with ABFR rates described in the recent literature. However, the reported incidence rates vary considerably. Some of which documents occurrence rates of up to 30 percent [3, 15, 16, 22, 23]. These wide range may result from different definitions of ABFR.

As shown in Table 4, this retrospective study is one of the largest investigating parameters affecting the incidence of ABFR after CP. Several classifications of ABFR have been described in the literature [4, 15, 24]. We focused our examinations on severe cases of ABFR corresponding to type II of the contemporary classification systems of Dunisch [4]. The stage of ABFR was defined as bone-flap thinning with resulting loss of brain protection and/or a cosmetically unacceptable appearance.

**Table 4:** Review of risk factors leading to ABFR found in scientific literature.

Author	Year	Number of performed CPs [N]	Study type	Age [years]	Preservation	Rate of severe ABFR	Risk factors of ABFR
Prolo et al. [22]	1979	53	prospective	Range: 7–69	Cryo (-20°C)	4.1%	Numbers of osteocytes
Iwama et al. [35]	2003	49	retrospective	Mean: 47.8 (range: 1–76)		2%	Gap between bone flap and skull edge
Grant et al. [16]	2004	40	retrospective	Mean: 9.3 (range: 0–19)	Cryo	Size of bone flap <75 cm^2^: 0% >75 cm^2^: 60%	Size of skull defect
Dunisch et al. [4]	2013	372	retrospective	Mean: 48.6 ± 18.4	Cryo (-80°C)	21.9%	Fragmentation of bone flap, ventriculoperitoneal shunting, lower age
Schuss et al. [32]	2013	254	retrospective	Mean: 45 ± 17	Cryo (-20°C)	4%	Fragmentation of bone flap, wound infection, cranioplasty timing
Ewald et al. [23]	2014	76	retrospective	Mean: 54.34 ± 10.73	Cryo (-80°C)	22.8%	
Stieglitz et al. [24]	2015	100	retrospective	Mean: 46.2 ± 18.0	Cryo (-80°C)	30.4%	
Brommeland et al. [2]	2015	87	retrospective	Median: 31 (range: 1–64)	Cryo (-20°C)	19.7%	Fragmentation of bone flap, lower age, Glasgow outcome scale, time of preservation
Zhang et al. [15]	2017	188	retrospective	Mean: 39.8 ± 13.13 (range: 15–67)	Cryo	6%	Location and fragmentation of bone flap, ventriculoperitoneal shunting
Ernst et al. [17]	2018	108	retrospective	Mean: 36 (range: 1–66)	In vivo (subcutaneous pocket)	9.3%	Fragmentation of bone flap, ventriculoperitoneal shunting, diabetes
Kim et al. [5]	2019	126	retrospective		Cryo (-80°C)	25%	cranioplasty timing
Current study	2019	303	retrospective	Mean: 51.2 ± 17.0	Cryo (-80°C)	3%	Karnofsky performance score, cranioplasty timing, (lower age)

Despite some attempts to study the subject, factors influencing ABFR development remain relatively unknown. Various studies have reported correlations between patient age [3, 25], multiple fractures [4, 15], bone flap localization [15], ventriculoperitoneal shunting [3, 26], and skull defect size [15, 16] with the development of ABFR.

Table 4 summarizes the results of our analysis in comparison to observed risk factors of the recent literature indicating that ABFR is fairly common in neurosurgery. In our study group a significant factor influencing the development of ABFR was found to be CP timing; the period between a DC and the subsequently performed CP (p = 0.008). According to our data, it seems that a later CP is associated with a lower risk of ABFR. These results are surprisingly in contrast to recent observations.[27] Other authors have suggested that an extended lapse of time between DC and CP actually leads to reduced bone-flap viability, which in turn may lead to failure in the remodeling of the bone matrix [28, 29]. However most studies have not detected any significant influence of the DC-CP time lapse on the risk of ABFR [1, 30, 31].

Furthermore, we observed that patients with a higher KPS showed a significant risk of developing ABFR. It is difficult to explain why patients with a KPS of 80 or higher should be more likely to develop ABFR than patients with a KPS of 60 or lower. The main difference between these groups is the fact that patients with a KPS of 80 or higher are consideredable to be self-reliant. In contrast, patients with a KPS of 60 or lower usually need professional help from caregivers. In the literature, we found no additional evidence regarding the KPS as an influencing factor. Interestingly, Brommeland et al. [2] found a high Glasgow outcome scale at time of CP associated with increased risk of ABFR. However, the authors assumed that undetected ABFR in patients in vegetative state biased the results.

**Fig 2:**
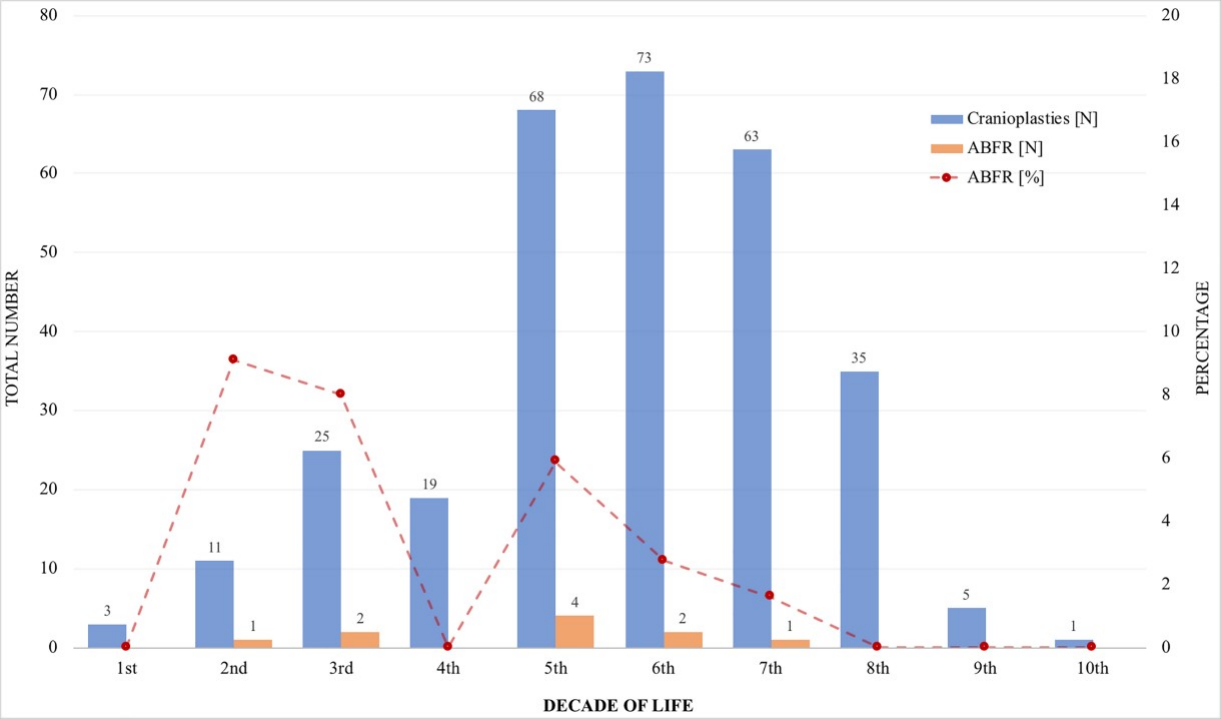
Absolute and relative number of ABFR in relation to decade of life.

According to our analyses, the age of the patient had no significant value for the development of ABFR, but statistical analysis shows that there is a trend. This tendency is also apparent in Fig 2 indicating that the younger the patient, the more likely it is that an ABFR will develop. Young age has previously been considered as a risk factor for ABFR in some studies [4, 21], although in a study by Kim et al. [27], no correlation between age and the risk of developing ABFR could be found.

In the present study, no significant correlation between multiple bone pieces and increased risk of ABFR was found. Our results are in line with the results of Grant et al. [16]. Using bone flaps that have multiple fractures has previously been considered a high risk for the occurrence of ABFR. Multiple fractured bone-flaps are assumed to be the underlying pathological mechanism [3, 4, 15].

The presence of a VPS was not associated with an increased ABFR rate in the current study, even though changes in intracranial pressure due to the siphon effect of the VP shunt have in some studies been suspected of leading to movement of the bone apparatus and increased bone resorption [4, 15]. In several studies, the presence of a VPS is accompanied by a significantly increased resorption rate of the bone flap that requires reoperation as opposed to the rate found in the absence of a VPS [7, 18, 30, 32].

The primary application of synthetic skull implants is a valid method for avoiding the risk of ABFR. In our opinion, however, the severely increased risk of infection and rejection, the higher costs, and the imperfection of the fit do not justify the primary implantation of alloplastic materials [13].

After a DC, removed bone flaps are preserved either in vivo or ex vivo [13, 15, 20, 33, 34]. Cryopreservation is the most common preservation method used [13, 15, 20, 35, 36]. Several authors have been able to show that the structural proteins necessary for the revitalization of the bone flap remained intact regardless of the duration of cryopreservation [16, 35]. In other studies, however, cryopreservation is associated with devitalization and an increased risk of ABFR [13, 37]. Since in the present study all bone flaps were cryopreserved only, we cannot make a comparison with alternative storage methods of the bone flap and their influence on ABFR. The most common alternative method of preserving bone flaps is to place them in vivo between the internal abdominal fat in a subcutaneous pocket [17].

### Limitation

A prospective multicenter study with a longer follow-up period could provide better results. A cost analysis regarding a reoperation due to ABFR and the fabrication of a synthetic skull implant was not performed in this study. A detailed cost analysis will be carried out in future studies.

## Conclusions

In the current study, CP timing and KPS were identified as factors significantly influencing the development of ABFR. Age is not significant but showed a trend in the development of ABRF in younger patients.

## Supporting information

S1 Data (XLS)
